# Enhanced Cancer Radiosensitization via Energy Transfer from Eu-Doped GdF_3_ Nanoparticles to Methylene Blue in X‑ray Photodynamic Therapy

**DOI:** 10.1021/acsami.5c10506

**Published:** 2025-09-04

**Authors:** Mileni M. Isikawa, Zeinaf Muradova, Toby Morris, João V. V. Lessa, Fernanda H. Borges, Rogéria R. Gonçalves, Ross I. Berbeco, Eder J. Guidelli

**Affiliations:** † Departamento de Física - FFCLRP, 28133 Universidade de São Paulo, Ribeirão Preto, SP 14040-901, Brazil; ‡ Department of Radiation Oncology, Brigham and Women’s Hospital, 1855 Dana-Farber Cancer Institute, Harvard Medical School, Boston, Massachusetts 02115, United States; § Department of Physics and Applied Physics, University of Massachusetts Lowell, Lowell, Massachusetts 01845, United States; ∥ Departamento de Química, Center for Nanotechnology and Tissue Engineering & Mater Lumen Laboratory − FFCLRP, 28133 Universidade de São Paulo, Ribeirão Preto, SP 14040-901, Brazil

**Keywords:** microfluidic, radioluminescence, lanthanides, energy transfer, radiotherapy, photodynamic therapy

## Abstract

We synthesized europium-doped gadolinium fluoride (GdF_3_:Eu) scintillating nanoparticles conjugated to methylene blue (MB) for singlet oxygen (^1^O_2_) generation in X-ray-induced photodynamic therapy (X-PDT). The impact of MB conjugation on GdF_3_:Eu nanoparticles (GdF@B) was analyzed, including size, polydispersity, and surface charge. Time-resolved photoluminescence analysis demonstrated that binding of MB to the nanoparticle surface is essential for enabling efficient resonant energy transfer (ET) from the GdF_3_:Eu core to the MB molecules. ET efficiencies ≥97% were obtained. ^1^O_2_ production exhibited energy- and dose-dependent behavior under varying radiation conditions. MTT assays demonstrated relative toxicity to LLC lung cancer mouse cells, while maintaining good tolerability in A549 human cancer cells. Clonogenic assays showed significant cytotoxicity in A549 cells only after X-ray exposure, confirming a reduced clonogenic survival. Survival fraction curves were analyzed using the linear-quadratic model, the sensitization enhancement factor, and the dose enhancement factor, highlighting the contributions of both the high atomic number of GdF_3_:Eu cores and ^1^O_2_ generation. The findings indicate that GdF@B enables deep-tissue ROS generation, overcoming light penetration limitations in traditional PDT. Additionally, these nanoparticles show potential to enhance radiotherapy efficacy in both conventional fractionated protocols and advanced radiosurgery techniques, offering a promising cancer treatment nanoplatform.

## Graphical abstract

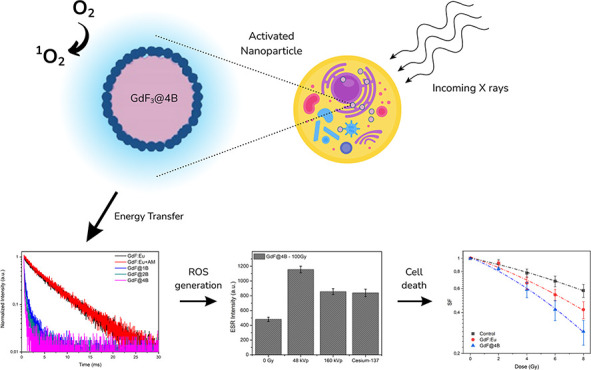

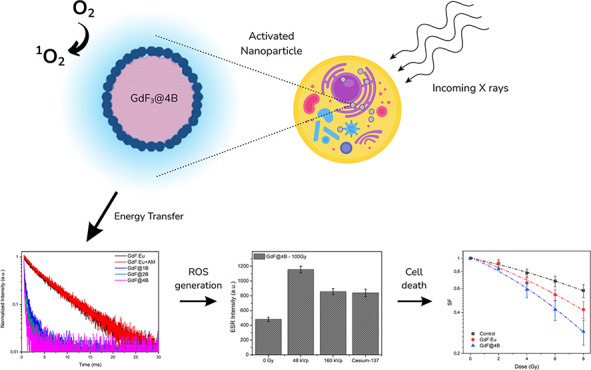

## 1 Introduction

Nanotechnology advances have significantly influenced the medical field, particularly through the development of novel cancer therapies. One promising area of research focuses on the application of scintillation nanoparticles (ScNPs) combined with radiotherapy to enhance cancer treatment efficacy while minimizing side effects. This treatment modality is callled X-ray activated photodynamic therapy (X-PDT).^1–4^

ScNPs emit light when exposed to ionizing radiation such as X-rays, making them suitable for both therapeutic and diagnostic applications. When integrated into cancer treatment protocols, ScNPs can substantially improve the precision and effectiveness of both traditional and alternative therapies, including X-PDT.^5^ Conventional photodynamic therapy (PDT) relies on visible light to activate photosensitizers (PS), producing reactive oxygen species (ROS) that destroy cancer cells.^6,7^

ROS exert cytotoxic effects in cancer therapy by inducing oxidative stress and disrupting key cellular processes. These reactive species cause direct damage to lipids, proteins, and DNA, leading to membrane disruption, protein dysfunction, and genomic instability.^8^ They also activate multiple signaling pathways, which can mediate cell survival, apoptosis, or inflammation, depending on context and ROS concentration. In mitochondria, they trigger permeability changes, releasing cytochrome c, and activating caspases that drive intrinsic apoptosis. Additionally, they can induce alternative cell death mechanisms such as ferroptosis (via lipid peroxidation and iron) and autophagy (as a protective or death response). They also cause cell cycle arrest through activation of DNA damage checkpoints and may promote cellular senescence.^9^

Despite the substantial ROS production by conventional PDT, its efficacy is limited by the shallow penetration depth (<1 mm) of visible light,^10^ restricting treatment to surface-level tumors. In contrast, X-rays can penetrate deeply into tissues, enabling broader and more effective treatment coverage through X-PDT.^11^ ScNPs function as internal light sources, emitting visible light upon X-ray exposure, which activates photosensitizers within deeper tissues. This results in effective ROS production and cancer cell death, even in tumors that are inaccessible to traditional PDT.

Several nanoparticles have been investigated for X-PDT, with each offering distinct advantages and limitations. Silicate-based NPs are promising due to their high chemical stability, biocompatibility, and good dispersibility in physiological conditions.^12–14^ However, their relatively low X-ray energy conversion efficiency limits their ability to generate ROS efficiently. Quantum dots (QDs), such as CdSe and CdTe NPs, provide strong X-ray absorption and size-tunable emission properties.^15,16^ Despite their optical advantages, cadmium-based QDs face cytotoxicity concerns, while research on heavy-metal-free scintillating QDs remains scarce.^17^

Oxide-based NPs, including TiO_2_ and ZnO, are widely studied for photodynamic therapy due to their strong photocatalytic activity and ROS generation upon irradiation.^16,18–20^ To enhance their X-ray energy conversion, researchers have explored doping with lanthanides.^21^ While these oxide NPs hold promise for theranostic applications, further optimization is needed for clinical translation.

Lanthanide-doped nanoparticles (LNPs) are particularly promising for X-PDT due to their unique optical and electronic properties. Materials such as Gd_2_O_3_ and Y_2_O_3_ doped with Eu^3^^+^ or Tb^3^^+^ exhibit efficient X-ray scintillation, enabling ROS activation through visible or near-infrared emissions.^22^ Fluoride-based LNPs like NaYF_4_ stand out for their excellent scintillation properties, high biocompatibility, and chemical stability.^12,22^ Additionally, Gd-containing NPs offer MRI contrast capabilities, furthering their potential as theranostic agents.^23,24^

Among Gd-based ScNPs, europium-doped gadolinium fluoride (GdF_3_:Eu^3^^+^) emerges as a strong candidate for X-PDT. Its high atomic number (*Z* = 64) enhances X-ray absorption and scintillation light emission.^24^ Eu^3^^+^ doping introduces strong red luminescence (590–720 nm), which falls within the therapeutic window, allowing deeper tissue penetration.^24^ This combination of high scintillation intensity, stability, and low toxicity makes GdF_3_:Eu^3^^+^ well-suited for combined therapeutic and diagnostic applications.^24^

Our group developed a microfluidic synthesis approach to grow GdF_3_:Eu^3^^+^ theranostic NPs with red and near-infrared scintillation for real-time monitoring of X-ray doses during X-PDT.^24^ Optimized microfluidic synthesis enhanced Eu^3^^+^ scintillation emission, while methylene blue (MB) conjugation in a core–shell structure prompted ^1^O_2_ generation under X-ray irradiation, achieving complete *E. coli* cell death.^24^ Since Eu^3^^+^ radioluminescence at 694 nm falls within the therapeutic window but is only partially absorbed by MB molecules, we explored its potential for in vivo dosimetry. Using pig skin and fat as tissue-mimicking models, we demonstrated that scintillation light could be detected through 16 mm of tissue, suitable for in vivo dosimetry in X-PDT procedures of breast cancer, for instance.^24^

In this study, we investigated the energy transfer from GdF_3_:Eu to MB to boost ROS generation for X-PDT. We also analyzed the impact of MB conjugation on nanoparticle size, polydispersity, and surface charge, complemented by time-resolved photoluminescence measurements to investigate the energy transfer mechanism. Additionally, ^1^O_2_ production was assessed under varying ionizing radiation beam energies, and MTT and clonogenic assays evaluated their cytotoxicity to human lung cancer cells (A549) with and without MB shells and in the presence and absence of X-ray irradiation.

## 2 Experimental Section

### 2.1 Materials

Europium­(III) nitrate pentahydrate (Eu­(NO_3_)_3_·5H_2_O, 99,99%), gadolinium­(III) nitrate pentahydrate hexahydrate (Gd­(NO_3_)_3_·6H_2_O, 99,99%), ethylene glycol (99%), 2,2,5,5-tetramethyl-3-pyrroline-3 carboxamide (TPC), and poly­(acrylic acid) (Mw = 1800) were obtained from Sigma-Aldrich. Ammonium fluoride (NH_4_F) and methylene blue (C_16_H_18_ClN_3_S) were purchased from Vetec and Teflon.

### 2.2 Synthesis of Europium-Doped Gadolinium Fluoride (GdF_3_:Eu) in the Microfluidic Reactor

GdF_3_:Eu cores were synthesized according to the following procedure: 19 mL ethylene glycol, 0.9 mmol Gd­(NO_3_)_3,_ and 0.1 mmol Eu­(NO_3_)_3_ were added into a three-neck flask and degassed at 100 °C for 10 min, and subsequently submitted to nitrogen flow for 10 min. This process was repeated three times to remove dissolved oxygen to prevent side reactions, and the heating was turned off. After the room temperature was achieved, 1 mL of 3 mmol of NH_4_F was injected into the reaction mixture and stirred for 5 min to form the nuclei. Then, the precursor solution was collected with a syringe and injected into a tubular microfluidic reactor, with 1 min of reaction time and temperatures of 194 °C, to allow particle growth, giving rise to GdF_3_:Eu nanostructures. The final solution was centrifuged for 30 min at 10,000 G (Eppendorf Centrifuge 5415D). The supernatant was discarded, and the pellet was resuspended in water. The dispersion was dried in an oven at 80 °C for 12 h.

### 2.3 Characterization

Powder X-ray diffraction was performed in a SIEMENS D5005 diffractometer with a copper cathode and a graphite monochromator to select the Kα_1_ emission region of copper (λ = 1.5418 Å). The potential at the source was 40 kVp, and the current was 40 mA. The X-ray patterns were obtained in the range between 20° and 90° (2θ) in a 0.2° /s step. The morphology and size of some samples were analyzed using transmission electron microscopy (TEM), JEOL-JEM-100 CXII. The samples were dripped onto a copper graticule covered with a conductive polymer and subjected to a drying process at room temperature. The particle size distribution and surface charge were measured by using the dynamic light scattering (DLS) technique with a Zeta-Sizer system (Malvern Instruments). The data were collected at a fixed angle at 173° and a fixed wavelength (633 nm/He–Ne laser). Thermogravimetric analyses of the samples were performed using a TA Instruments Q-600 system (Simultaneous DTA/TGA/DSC). The measurements were carried out under a synthetic air atmosphere with a heating rate of 10 °C/min up to a final temperature of 800 °C.

Radioluminescence (RL) measurements were performed with an X-ray tube (Magnum, Moxtek, USA), operating at 48 kVp and 0.2 mA. Emission was collected by an optical fiber coupled to an Ocean Optics spectrometer (USB4000) with an integration time of 30 s. The energy transfer efficiency estimated by the relative RL intensity was calculated by the same method employed for fluorescence, following the equation:^25^

$$E_{\mathrm{RL}}=1-\frac{{\mathrm{RL}}_{\mathrm{DA}}}{{\mathrm{RL}}_{D}}$$ 1

where RL_DA_ is the relative RL intensity (area) of the donor (GdF_3_:Eu) in the presence of the acceptor (MB), and RL_D_ is the relative RL intensity (area) of the donor in the absence of the acceptor.

Photoluminescence spectra (PL) and the emission decay curves were measured using a Fluorolog 3 Horiba Scientific spectrofluorometer (model FL3-22), equipped with double monochromators on both excitation and emission. A 450 W Xe lamp was employed as the excitation source for the emission/excitation spectra, while a 450 W Xe pulsed lamp was used for lifetime measurements. A Hamamatsu R928 photomultiplier was used for detection in the visible range. The lifetime measurements were initiated with a delay of 45 μs, which ensured that any residual lamp pulse had fully decayed and did not interfere with the analysis of the Eu^3+^ emission. In this regime, the instrument response has negligible influence on the measured decay times.

The energy transfer efficiency estimated by the lifetime measurements was calculated by the following equation:^25^

$$E_{\tau }=1-\frac{\tau_{\mathrm{DA}}}{\tau_{D}}$$ 2

where τ_DA_ is the lifetime of the donor (GdF_3_:Eu) in the presence of the acceptor (MB), and τ_D_ is the lifetime of the donor in the absence of the acceptor.

### 2.4 X-ray Activated Photodynamic Therapy

#### 2.4.1 Conjugation of Methylene Blue (MB) on the GdF_3_:Eu Nanoparticles

To conjugate methylene blue (MB) on the GdF_3_:Eu nanoparticles surfaces, 0.01 mmol L^–1^ MB and 20 wt % of poly­(acrylic acid) (PAA) stock solutions were prepared. The conjugated MB and PAA were produced in the following steps: 1 mL of the PAA solution was mixed with 5 mg of nanoparticles, and after 20 min, it was centrifuged at 10,000 G for 30 min. The supernatant was removed and resuspended in 1 mL of Milli-Q water for 5 min to eliminate weakly bound PAA molecules. The sample was again centrifuged with the same parameters, the supernatant was removed, the sample was resuspended in 1 mL of the MB stock solution, and the mixture was kept under stirring for 20 min. After centrifuging, the supernatant with MB was discarded, and the pellet was resuspended in Milli-Q water or PBS (phosphate buffer saline, pH = 7.4) solution, resulting in GdF_3_:Eu covered with 1-time of PAA/MB, named as GdF_3_@1B core–shell nanoparticles. This procedure was repeated several times to produce nanoparticles and improve the coverage with MB. The conjugation procedure was repeated 1, 2, 4, and 6 times, named GdF_3_@1B, GdF_3_@2B, GdF_3_@4B, and GdF_3_@6B, respectively. The 1-time PAA/MB was investigated with different MB concentrations (1, 0.1, 0.01, and 0.001 mM) to investigate the MB concentration effect.

#### 2.4.2 Measurement of Singlet Oxygen Production

A 1 mol·L^–^^1^ stock solution of the spin trap TPC in ethanol was prepared. Then, 60 μL of this solution was added to dispersions containing bare GdF_3_:Eu and GdF_3_@4B. The dispersion was divided into two equal volumes: one was kept in the dark as a control, while the other was irradiated with 100 Gy of X-rays under varying conditions. The ionizing radiation beams were produced by several sources including a 48 kVp (Magnum, Moxtek, USA), and a 160 kVp (Isovolt Titan E-160M-2 GE) X-ray tubes, and a gama emitting Cesium-137 source. Doses ranged from 0 to 120 Gy.

Electron spin resonance (ESR) spectra were recorded at room temperature using a JEOL JES-FA 200 spectrometer (9.5 GHz). The measurement parameters were 2 mW microwave power, 335.6 mT central field, 15 mT sweep width, 2 min sweep time, 2 mT modulation amplitude, 10,000 amplifier gain, and five scans for signal averaging in each sample. These experiments were performed in triplicate. Singlet oxygen production was quantified by calculating the ratio of the peak-to-peak intensity of the TPC ESR spectrum for irradiated samples to that of nonirradiated samples kept in the dark.

### 2.5 Cytotoxicity of Nanoparticles

#### 2.5.1 Cell Viability Using the MTT Assay

The viability of human nonsmall cell lung cancer A549 cells (ATCC CCL-185, USA) and murine LL/2-Fluc-Neo/eGFP-Puro (LLC) cells (CL073-STAN, USA) treated with bare GdF_3_:Eu and GdF@4B nanoparticles was evaluated using the MTT assay. A549 and LLC cells were maintained in RPMI 1640 media (Gibco, Invitrogen, USA) and DMEM-GlutaMax (Life Technologies, USA), respectively, containing 10% heat-inactivated fetal bovine serum (Invitrogen, USA) and 1% penicillin-streptomycin (Invitrogen, USA) cocktail in a 37 °C humidified incubator with 5% CO_2_. The cells were seeded in 96-well plates at a 10^4^ cells/well density. GdF_3_:Eu and GdF_3_@4B nanoparticles were initially dispersed in nonsupplemented RPMI 1640 medium at a concentration of 5 mg/mL. Subsequent dilutions (0.075, 0.125, 0.25, 0.75, and 1.25 mg/mL) were prepared using RPMI 1640 medium supplemented and used to treat the cells after 24 h. Cells treated with bare RPMI 1640 medium with supplements were used as controls. All treatments were performed in triplicate. After 48 h, 100 μL of the 3-(4,5-dimethylthiazol-2-yl)-2,5-diphenyltetrazolium bromide (MTT, Sigma-Aldrich, USA) solution at 5 mg/mL was added to each well and maintained for 4 h. Formazan crystals were then formed by mitochondrial reduction of MTT, and 100 μL of DMSO (Invitrogen, USA) was used to solubilize the purple formazan crystals. The absorbance of the formazan product was measured at a 570 nm wavelength (λ) by an ELISA Reader (Clariostar). Cell viability (%) was calculated by the ratio of the absorbance of samples treated with nanoparticles to the absorbance of the control samples (without nanoparticles). The data represent the average of triplicate measurements from three independent experiments. Two-way ANOVA tests were performed to analyze the MTT assay results.

#### 2.5.2 Cell Reproductivity by Clonogenic Assay

The clonogenic assay was performed to determine the reproductive capacity of A549 after ionizing radiation treatment. The A549 cells were maintained in RPMI 1640 medium containing 10% heat-inactivated fetal bovine serum and a 1% penicillin-streptomycin cocktail in a 37 °C humidified incubator with 5% CO_2_. The cells were seeded with different densities in 6-well plates: for doses of 0 and 2 Gy, the density was 5 × 10^2^ cells/well, and for 4, 6, and 8 Gy, the density was 8 × 10^2^ cells/well. After 24 h, the cells were treated with bare GdF_3_:Eu and GdF@4B at a 0.075 mg/mL concentration dispersed in RPMI 1640 medium with supplements. The control group (without nanoparticles) was replaced with RPMI 1640 medium. The plates were incubated for 48 h and then irradiated with 2, 4, 6, and 8 Gy with a Varian X-ray tube of a small animal radiation research platform at 220 kVp and 13 mA (Xtrahl, Inc., Suwanee, GA). Four hours after irradiation, the medium was replaced, and the medium in the wells was replaced every 4 days until colonies had at least 50 cells. After colony formation, the plates were fixed with ethanol (70% v/v) for 24 h at 4 °C. After that, the wells were stained with 0.1% crystal violet and 6% glutaraldehyde for 24 h. Finally, the plates were washed three times with Milli-Q water and dried at room temperature to count. Measurements were performed in triplicate, and quantification was conducted using an in-house ImageJ colony counter macro. Two-way ANOVA tests were performed to analyze the clonogenic assay results.

## 3 Results and Discussion

### 3.1 Characterization of GdF_3_:Eu

To characterize the GdF_3_:Eu nanoparticles, we analyzed their morphological, structural, and optical properties. Figure 1a,c displays TEM images along with the corresponding size distribution histograms of freshly synthesized and self-assembled GdF_3_:Eu nanoparticles (Figure 1b,d, respectively). In this study, “GdF_3_:Eu” refers to nanoparticles characterized immediately after synthesis, while “self-assembled” denotes samples examined one week postsynthesis. A key distinction between the two samples was their shape; immediately after synthesis, the GdF_3_:Eu nanoparticles exhibited a rhombus morphology, whereas the self-assembled samples developed a flower-like structure. Furthermore, the average diameter increased from 13 ± 6 nm in freshly synthesized samples to 44 ± 13 nm in the self-assembled state.

**Figure 1:**
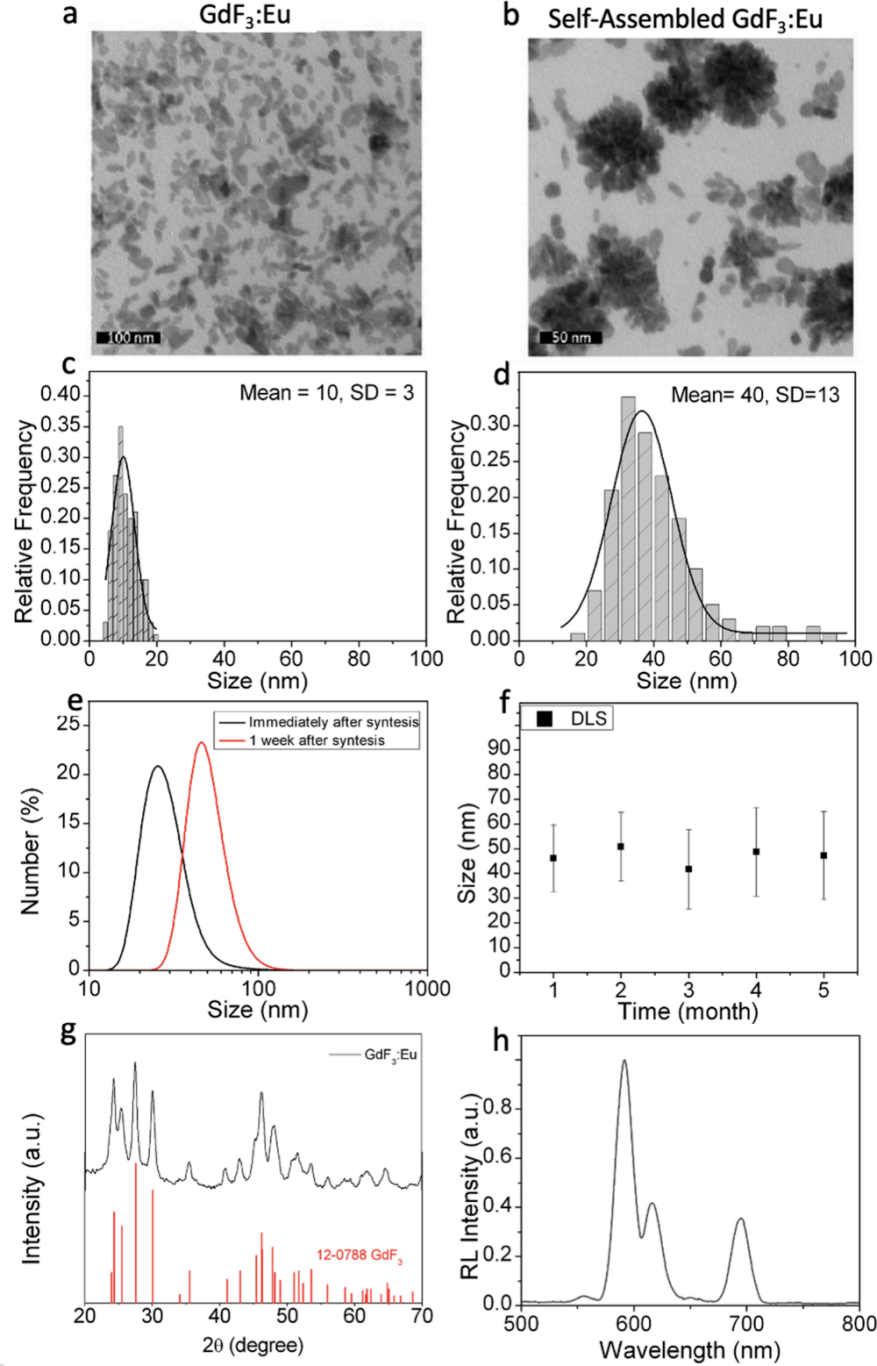
Characterization of GdF_3_:Eu nanoparticles. (a) TEM images of GdF_3_:Eu nanoparticles immediately after synthesis and (b) self-assembled GdF_3_:Eu nanoparticles 1 week postsynthesis. (c,d) Size-distribution histograms calculated from TEM images a and b, respectively. (e) DLS measurements comparing hydrodynamic sizes of freshly synthesized and self-assembled GdF_3_ nanoparticles. (f) Time-dependent size measurements obtained from DLS. (g) X-ray diffractograms showing the characteristic orthorhombic phase (12-0788) of GdF_3_:Eu. (h) Radioluminescence spectra of the as prepared samples.

The DLS measurements presented in Figure 1e indicate a hydrodynamic size of 28 ± 10 nm for GdF_3_:Eu nanoparticles and 48 ± 14 nm for self-assembled nanoparticles. The differences in size distribution between DLS and TEM measurements for freshly synthesized samples can be attributed to variations in shape; while TEM provides size estimations based on the actual morphology of the nanoparticles, DLS approximates the size assuming a spherical geometry. Conversely, for self-assembled nanoparticles, which displayed a roughly spherical shape, the size measurements from both techniques exhibited minimal differences (<5 nm). Furthermore, the polydispersity index (PDI) of both samples was below 0.2, indicating low polydispersity, consistent with the monodisperse shapes observed in the TEM images. Additionally, the zeta potential, which is related to colloidal stability and surface charge,^26^ increased from 37 ± 6 mV for freshly synthesized GdF_3_:Eu nanoparticles to 45 ± 6 mV for self-assembled nanoparticles, suggesting enhanced stability following the self-assembly process. DLS analysis investigated the colloidal stability for months, as shown in Figure 1f. The hydrodynamic size remained constant for at least 5 months.

Figure 1g presents the XRD patterns for GdF_3_:Eu, revealing an orthorhombic phase (12-0788), which is known to be the most thermodynamically stable structure for GdF_3_:Eu.^27^ The crystallite size, calculated using the Scherrer equation,^28^ was 15.5 nm, which closely matches the values obtained from TEM images of GdF_3_ nanoparticles, indicating the formation of monocrystalline structures. The optical properties of the nanoparticles were investigated using radioluminescence (RL), shown in Figure 1h, and photoluminescence (emission spectra), shown in Figure S1. The GdF_3_:Eu matrix itself exhibited no luminescence, whereas the Eu^3+^ emission was clearly observed, corresponding to the characteristic transitions: ^5^D_0_ → ^7^F_0_ (554 nm), ^5^D_0_ → ^7^F_1_ (594 nm), ^5^D_0_ → ^7^F_2_ (614 nm), ^5^D_0_ → ^7^F_3_ (650 nm), and ^5^D_0_ → ^7^F_4_ (697 nm).^29^ The presence of Eu^3+^ emission in both the RL and photoluminescence spectra confirms the successful doping of Eu^3+^ into the GdF_3_:Eu structure. Additionally, the excitation spectra shown in Figure S1 display several peaks, with multiplet around 272 nm, corresponding to Gd^3+ 8^S_7/2_ → ^6^I_J_ transition, and at 394 nm, corresponding to Eu^3+ 7^F_0_→^5^L_6_ transition, both of which generate emissions at 594 nm. It is important to note that Figures 1g,h, S1, and S2 show no changes in the structural (XRD) or optical (RL and PL spectra) properties of GdF_3_:Eu samples, both immediately after synthesis (pristine) and after 1 week of characterization. The difference was restricted to the size and morphology (Figure 1a–d).

### 3.2 Conjugation of Methylene Blue in GdF_3_:Eu Nanoparticles

Once suitable nanoparticles with small size and intense scintillation were obtained, we explored the possibility of using Eu^3^^+^ radioluminescence (RL) emissions for X-PDT. For effective X-PDT, the ScNPs must be conjugated to a PS whose absorption spectrum overlaps with the ScNP emission (Figure S3). In the case of our GdF_3_:Eu nanoparticles, MB) exhibits good spectral overlap with the Eu^3^^+^ emission bands. MB is a clinically relevant photosensitizer capable of generating ROS and has demonstrated strong potential in cancer and antimicrobial photodynamic therapy.^30^ However, direct excitation of MB by X-rays is inefficient, since X-rays do not effectively excite its molecular orbitals. When combined with scintillating nanoparticles, MB shows promise for X-PDT by enabling indirect excitation via Eu^3^^+^ emissions.^31^ Specifically, the Eu^3^^+^ emissions at approximately 594 and 614 nm (corresponding to the ^5^D_0_ → ^7^F_1_ and ^5^D_0_ → ^7^F_2_ transitions, respectively) overlap well with MB’s absorption bands at 614 and 664 nm (Figure S3).

The conjugation occurred via electrostatic bonding. However, since both GdF_3_ nanoparticles and MB possess a positive charge, poly­(acrylic acid) (PAA), which carries a negative charge, was used to modify the nanoparticle surface to a negative charge. Once the surface charge was altered, the sample was mixed with a MB solution of varying MB concentrations and subsequently centrifuged and washed to produce GdF_3_:Eu nanoparticles with one deposition cycle of MB (GdF@1B). It is worth noting that, from here until the end of the manuscript, the subscript “3” and term “Eu” will be omitted when referring to the core–shell nanoparticles solely for simplification purposes, but all the conjugated samples employed GdF_3_:Eu.

The initial optimization focused on the influence of the MB concentration on the conjugation process. Hydrodynamic size measurements of the nanostructure (Figure 2a,b) revealed that at higher MB concentrations, GdF_3_:Eu nanoparticles tended to agglomerate, increasing both polydispersity and hydrodynamic size, with values shifting from 63 ± 35 nm and a PDI of 0.230 to 269 ± 164 nm and a PDI of 0.504. Despite this, the zeta potential remained negatively charged at approximately −20 mV due to the presence of PAA at the GdF_3_:Eu surface (Figure 2c).

**Figure 2:**
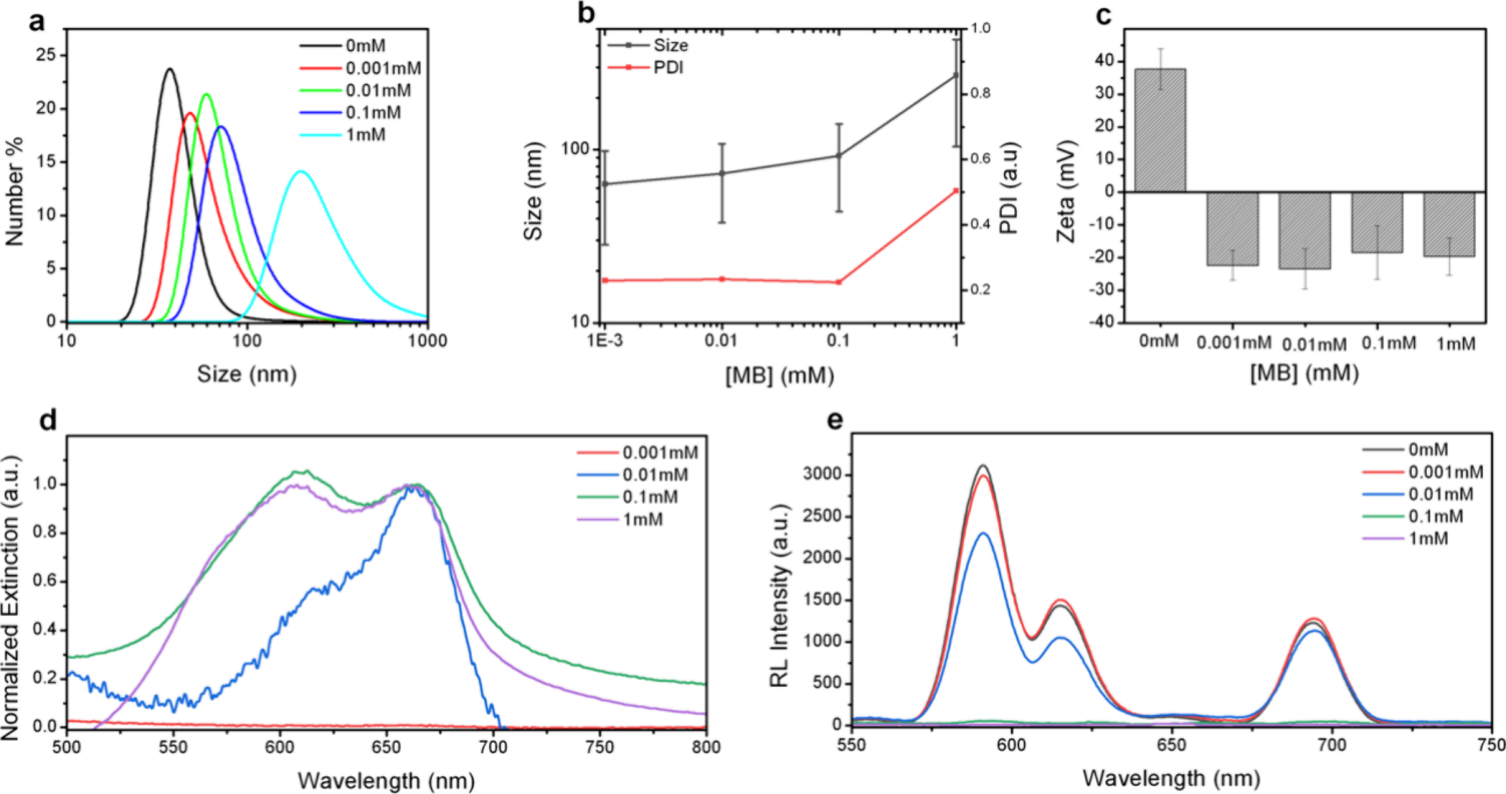
(a) DLS measurements, (b) size distribution and polydispersity index (PDI), and (c) zeta potential measurements for GdF_3_@1B nanoparticles produced at varying MB concentrations. (d) Normalized absorption spectra* and (e) Radioluminescence (RL) spectra for GdF_3_@1B nanoparticles under different MB concentrations. * The absorption spectrum of GdF_3_@1B synthesized at MB concentration of 0.001 mM was not normalized, as the low absorbance values resulted in significant noise, obscuring the spectral details observed in other samples.

UV–vis and RL spectra of the GdF@1B produced at a concentration of 0.001 mM evidenced that MB adsorption over the nanoparticle surface was not effective due to the very low absorbance values (Figure 2d) obtained and because RL intensity was not significantly quenched (Figure 2e). Upon higher MB concentrations, the formation of a dimer structure is suggested due to the notorious blue shift with an increased peak intensity at 605 nm and a decreased peak intensity at 664 nm (Figure 2d).^32,33^ At a MB concentration of 1 mM used for deposition onto GdF_3_:Eu nanoparticles, the formation of trimeric species is indicated by the appearance of an additional absorption band around 580 nm. Using known extinction coefficients and the Beer–Lambert law, the concentrations of monomeric and dimeric MB species adsorbed onto the bare GdF_3_:Eu nanoparticle surface as a function of the MB stock concentration were estimated (Figure S4a). The adsorbed concentrations of both species increased with the MB stock concentration used during the deposition process. However, the increase was more pronounced for the monomer. This suggests a preferential interaction of MB monomers with the nanoparticle surface. The simultaneous increase in nanoparticle size and monomer adsorption supports the hypothesis that MB monomers form a surface coating layer, contributing to the observed size growth. Additionally, partial nanoparticle aggregation mediated by MB interactions (e.g., π–π-stacking or electrostatic bridging) may also play a role in the observed size changes.

Along with enhanced dimer formation, increasing the MB concentration to produce GdF@1B also led to a consistent decline in RL intensity (Figure 2e), indicating greater adsorption of MB onto the GdF_3_:Eu surface. Therefore, considering the hydrodynamic size and PDI, the MB concentration of 0.01 mM was selected as optimal for growing an MB shell over the GdF_3_:Eu surface for the subsequent experiments.

The second optimization step, following the selection of the optimal MB concentration (0.01 mM) to grow the MB shell, focused on evaluating the influence of the number of PAA/MB deposition cycles. This process involved sequentially coating the GdF_3_:Eu nanoparticles with layers of PAA, followed by MB, and repeating this cycle multiple times to ensure complete surface coverage and the formation of a core–shell structure. Each cycle of PAA/MB deposition is termed GdF@nB, where n denotes the number of deposition cycles of the GdF_3_:Eu core, varying from 1 to 6 (Figure 3a).

**Figure 3:**
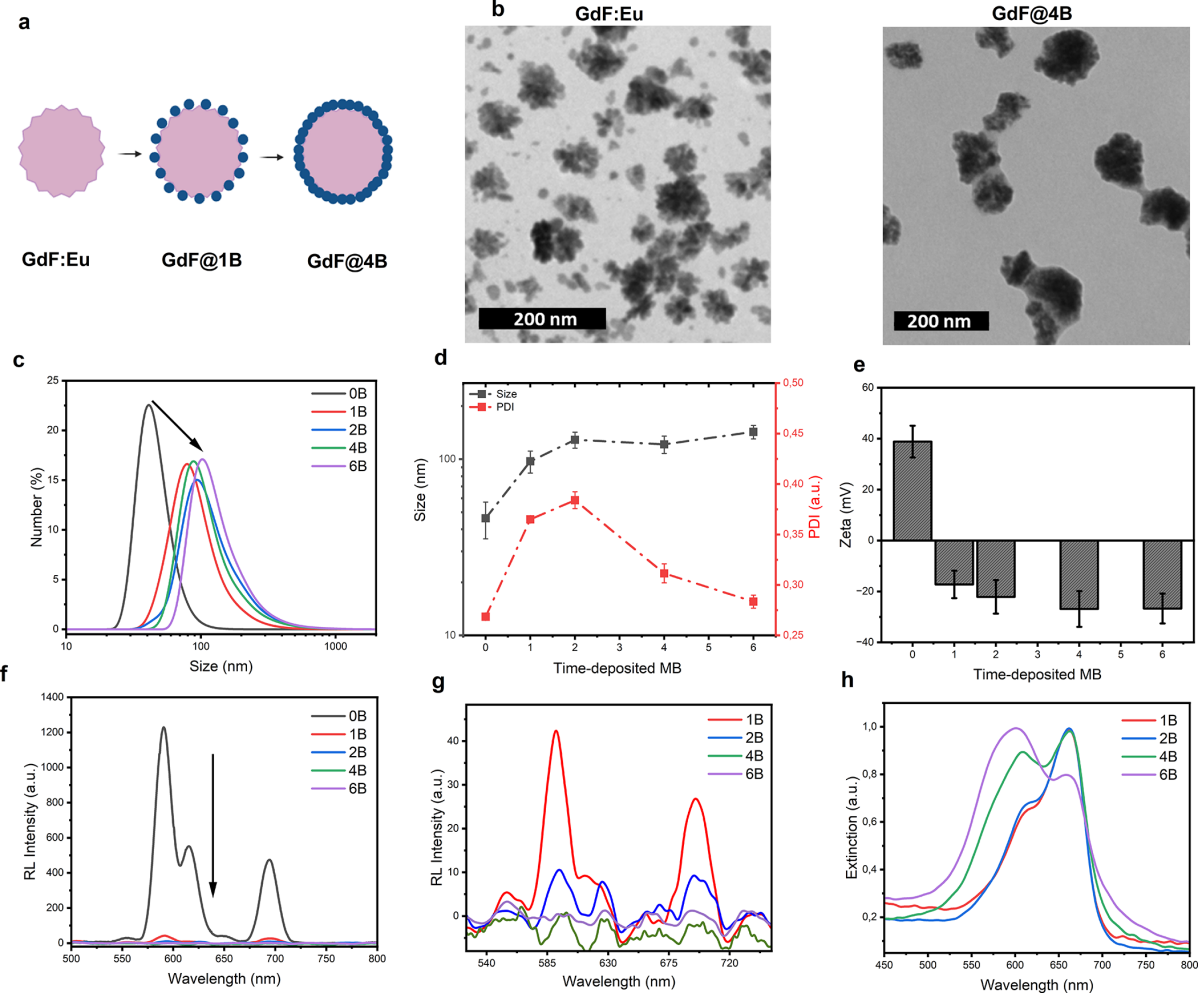
(a) Schematic representation of the growth of PAA/MB bilayers on ScNP (GdF@nB). TEM images of bare GdF_3_:Eu (GdF@0B) (b) and GdF@4B nanoparticles. (c) DLS size distribution, (d) size and polydispersity index (PDI), (e) zeta potential measurements, and (f) radioluminescence (RL) spectra for GdF@0B to GdF@6B (g) RL intensity as a function of the number of deposition cycles, evidencing a significant RL quenching. (h) Normalized absorption spectra for varying bilayer formations, evidencing dimer formation for increased MB deposition cycles.

Before PAA/MB deposition, TEM images revealed a mixture of two types of nanoparticles: smaller particles and larger self-assembled particles. Following PAA/MB deposition, only a single type of nanoparticle was observed, indicating that the self-assembly and agglomeration process was favored (Figure 3b). Additionally, after PAA/MB deposition, a thin shell was visible around the GdF_3_:Eu nanoparticles (Figure 3b), suggesting the successful formation of a coating layer composed of PAA or MB. DLS analysis (Figure 3c) showed that hydrodynamic size increased with successive PAA/MB depositions, from 45 ± 6 nm (GdF_3_:Eu) to 103 ± 53 nm (GdF@4B), with the size stabilizing around 100 nm after multiple depositions. Additionally, the PDI (Figure 3d) exhibited a decreasing trend with PAA/MB deposition cycles, indicating an increasing tendency toward monodispersity with each layer added. Zeta potential measurements (Figure 3e) indicated a positive surface charge for bare GdF_3_:Eu nanoparticles (38 ± 6 mV) and a negative charge for PAA/MB-coated nanoparticles (−26 ± 8 mV), reflecting the negatively charged characteristics of PAA (Figure 2c).

Thermogravimetric analysis (TGA) revealed a significantly higher mass loss in the region corresponding to the decomposition of organic components in the functionalized GdF_3_ samples compared to that of the bare GdF_3_:Eu (Figure S5). The conjugated nanoparticles exhibited a mass loss of approximately 10, 12.5, and 15%, for the samples GdF@1B, GdF@2B, and GdF@4B, respectively, further confirming the adsorption of methylene blue (MB) and polyacrylate (PAA) onto the nanoparticle surface (Figure S5). The progressive increase in mass loss upon increasing the number of deposition cycles indicates a higher incorporation of organic material in the functionalized samples. However, despite this result, it is not possible to individually quantify the contribution of methylene blue, as its thermal degradation occurs simultaneously with that of PAA, making it difficult to distinguish their respective contributions.

The RL signal was significantly reduced following PAA/MB deposition (Figure 3f,g), with each additional PAA/MB deposition cycle leading to further reductions. Specifically, the RL intensity for GdF@1B decreased around 20-fold, and for GdF@2B, it was reduced 34-fold (Figure 3g). In the cases of GdF@4B and GdF@6B, quenching of the RL signal is considered complete once the RL intensity is at the noise level. As revealed by Figure 3h, the UV–vis spectra for GdF@nB exhibited changes in the absorption band based on the amount of MB deposition. At higher MB deposition levels, the formation of dimeric species was favored, as evidenced by a blue shift in the UV–vis spectra. Consequently, monomeric MB was predominantly observed for GdF@1B and GdF@2B, while dimeric species became prominent in GdF@4B and GdF@6B (Figure 3h). The concentrations of monomeric and dimeric MB adsorbed onto the nanoparticles were estimated by using known extinction coefficients and the Beer–Lambert law (Figure S5). As the amount of deposited MB increased, the concentration of dimeric MB steadily increased. In contrast, monomer concentration initially increased from GdF@1B (∼1.2 μM) to GdF@2B (∼2.3 μM) but subsequently decreased and remained constant for GdF@4B and GdF@6B (∼2.0 μM). This suggests that beyond a certain MB loading, surface saturation or dimerization limits further monomer adsorption. Interestingly, no significant change in nanoparticle size was observed for GdF@4B and GdF@6B despite increased MB deposition and dimer formation. This suggests that only monomeric MB adsorption contributes appreciably to nanoparticle size growth, likely due to the formation of a surface coating. At higher MB loadings, surface saturation and enhanced dimerization may limit further monomer binding, resulting in a plateau in the size measurements. The dimeric MB species may adsorb in a compact conformation that does not significantly affect the hydrodynamic diameter.

The decrease in the RL signal and the formation of MB dimers indicate a large amount of MB molecules attached to the GdF_3_:Eu nanoparticles' surfaces. For MB dimers, the increased overlap between MB absorption and europium emission could enhance type I ROS production due to greater MB excitation, thereby increasing the overall ROS yield.

To investigate if resonant energy transfer is occurring in our nanoconjugates, time-resolved photoluminescence measurements were acquired for the bare GdF_3_:Eu suspended in water, bare GdF_3:_Eu suspended in MB solution (0.01 mM), and for the GdF@1B, GdF@2B, and GdF@4B. No difference was observed when comparing decay times of the bare GdF_3_:Eu suspended in water and suspended in the MB solution, suggesting that the energy transfer from the GdF_3_:Eu nanoparticles to MB molecules in solution is negligible (Figure 4a). However, for the GdF@nB core–shell nanoparticles, a significant and consistent decrease in the lifetimes is observed. Fitting the curves by a monoexponential decay, we obtained τ = (4.87 ± 0.01) ms for bare GdF_3_:Eu suspended in water, τ = (4.78 ± 0.02) ms, for bare GdF_3_:Eu suspended in MB solution, τ = (0.48 ± 0.01) ms for the GdF@1B, τ = (0.25 ± 0.01) ms for the GdF@2B, and τ = (0.11 ± 0.01) ms for the GdF@4B. This result reveals that resonant energy transfer only occurs when the MB is bound to the GdF_3_:Eu surface. Because both GdF_3_:Eu NPs and MB molecules present a positive charge, when bare GdF_3_:Eu is suspended in the MB solution, they will be too far apart due to electrostatic repulsion, hindering energy transfer.

**Figure 4:**
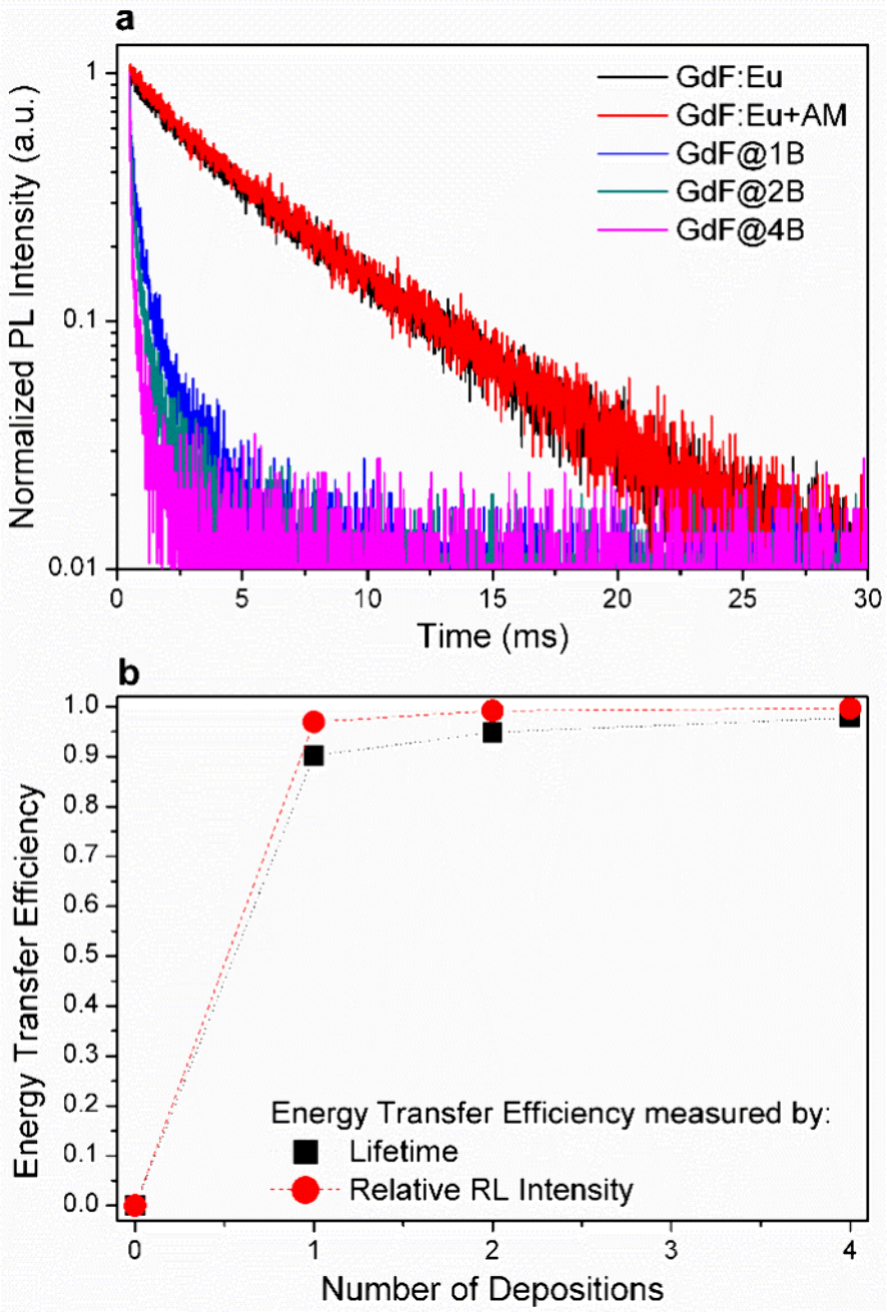
(a) Photoluminescence decay curves for the bare GdF_3_:Eu suspended in water, bare GdF_3:_Eu suspended in MB solution (0.01 mM), and for the GdF@1B, GdF@2B, and GdF@4B. Samples were excited at 394 nm and the luminescence emission collected at 592 nm. (b) Energy transfer efficiencies calculated by eqs 1 (relative RL intensity) and 2 (lifetime).

Figure 4b depicts the energy transfer efficiency as a function of the number of MB deposition cycles, calculated by the PL lifetime and relative RL intensity equations (eqs 1 and 2), revealing good agreement between the two methodologies. For GdF@4B, energy transfer efficiencies of up to 97.7 and 99.6% were achieved based on the lifetime and relative RL intensity measurements, respectively. The slightly higher energy transfer efficiency as measured by the relative RL intensity method is probably associated with the lower sensitivity of the CCD employed for RL measurement compared to the PMT tube employed for the lifetime measurements. In this sense, the 97.7% efficiency obtained via the lifetime method is considered the most reliable value. Because eq 2 can only be used when the donor–acceptor pairs are separated by a fixed distance, we did not calculate for the sample consisting of bare GdF_3_:Eu suspended in MB solution because, in this case, there is no single fixed donor–acceptor distance.

Considering that RL quenching was observed for GdF@4B and that energy transfer efficiencies calculated by eqs 1 and 2 are 99.6 and 97.7%, respectively, this sample was selected for further experiments. For this sample, the estimated MB concentration is ∼2.0 μM for monomers and ∼1.5 μM for dimers.

#### 3.2.1 Measurement of Singlet Oxygen Production

To evaluate the potential of GdF_3_:Eu nanoparticles for X-PDT, we used ESR spectroscopy with the spin-trap TPC, known for its high specificity in detecting ^1^O_2_.^34,35^ In this experiment, both bare GdF_3_:Eu and GdF@4B nanoparticles were exposed to X-rays, and ^1^O_2_ production was assessed by comparing the intensity of the TPC-ESR spectra of X-ray-irradiated samples with that of nonirradiated controls.

The first parameter analyzed was the influence of the X-ray energy on ^1^O_2_ production for GdF@4B nanoparticles. The highest ESR intensity was observed at a lower X-ray energy of 48 kVp (10 keV effective energy), with a progressive decrease in intensity as the X-ray energy increased to 160 kVp (42 keV effective energy) (Figure 5a). This behavior can be attributed to the predominant interaction mechanism for X-ray energies below 100 keV, which is photoelectric absorption by GdF_3_:Eu cores.^36^ As the X-ray energy increases, the probability of photoelectric interactions decreases, resulting in reduced ^1^O_2_ generation at higher energies.

**Figure 5:**
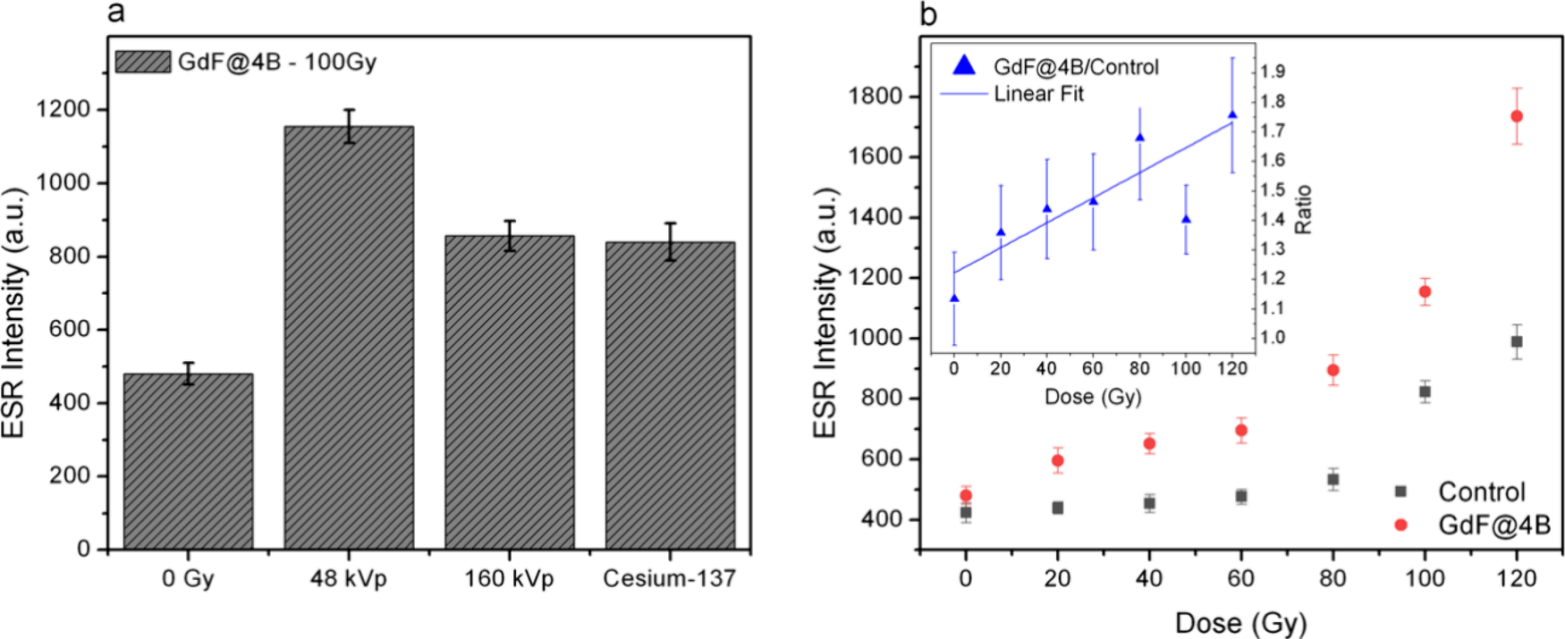
(a) TPC-ESR intensity measured in the presence of GdF@4B nanoparticles at varying beam energies. (b) Comparison of TPC-ESR intensity for control (GdF_3_:Eu) and GdF@4B nanoparticles across different X-ray doses. The inset depicts the GdF@4B/control ratio, suggesting that ^1^O_2_ production is linearly proportional to the dose.

Optimal ^1^O_2_ generation at 48 kVp is linked to enhanced X-ray–nanoparticle interactions, leading to the emission of a greater number of scintillation photons, particularly from Eu emission within the GdF_3_ matrix. These scintillation photons are effectively absorbed by the PS molecules, thereby boosting the level of ^1^O_2_ production. This phenomenon highlights the role of X-ray energy tuning in maximizing energy efficiency for photodynamic applications.

Interestingly, even with high-energy photon beams, such as the 642 keV γ rays emitted from a Cesium-137 source, a 2-fold increase in TPC signal was observed, indicating efficient ^1^O_2_ production even for high-energy beams. This suggests that the GdF@4B system retains a level of efficacy with high-energy radiation, demonstrating its potential applicability in radiotherapy protocols, which frequently employ high-energy photon beams to achieve deeper tissue penetration. It is noteworthy that samples irradiated with 160 kVp (42 keV effective energy) and 662 keV exhibited similar TPC-ESR signal intensities. However, it should be emphasized that the TPC signal intensity serves as an indirect indicator of ^1^O_2_ production, rather than a precise measure of its absolute concentration. Further systematic studies are needed to elucidate how ionizing radiation influences ^1^O_2_ detection via TPC. Nevertheless, these findings highlight the versatility of GdF@4B nanoparticles in enabling localized and controllable ^1^O_2_ generation under various photon beam energies, presenting promising potential for improving deep-tissue cancer treatment through the combined effects of radiotherapy and photodynamic therapy.

We then evaluated the influence of the X-ray dose on ^1^O_2_ generation using an X-ray beam energy of 48 kVp. The control group consisted of GdF_3_:Eu nanoparticles without methylene blue (MB) to assess radiation-induced TPC signals. As expected, the ESR intensity increased even in the control group (Figure 5b). This is probably associated with the radiolysis of water in the presence of dissolved oxygen.^37,38^ This background ROS detection serves as a baseline for comparison.

Although GdF_3_:Eu (control) increased by 2-fold the TPC signal, the GdF_3_@4B sample exhibited a significantly greater increase in ESR intensity compared to the control group. This pronounced gain in ESR intensity suggests that MB molecules effectively absorbed the light emitted from the GdF_3_:Eu cores and produced ^1^O_2_. The layered PAA/MB coating on the nanoparticles likely facilitated efficient energy transfer, leading to the excitation of the MB molecules and subsequent generation of singlet oxygen upon X-ray irradiation. It is important to note that the TPC-ESR intensity increases upon increasing the dose for both the control and GdF@4B samples. By calculating the GdF@4B/Control TPC-ESR signal ratio (Figure 5b inset), it is clear that the signal increase associated with ^1^O_2_ production is linearly proportional to the doses in the dose range tested. This result underscores the potential of GdF@4B as a nanoplatform for X-ray-induced photodynamic therapy (X-PDT), where precise and localized ^1^O_2_ generation is critical for therapeutic efficacy. The findings further support the synergistic role of scintillation-driven photosensitizer activation in maximizing ROS production for potential cancer treatment applications.

In fact, the mechanism behind singlet oxygen generation upon X-ray activation in nanoparticle-based systems remains multifaceted and energy-dependent. Nanoparticle design plays a critical role, as it can enhance energy absorption from the X-ray beam and subsequently improve activation of the photosensitizer bound to the nanoparticle surface.^39^ Although energy transfer from scintillating nanoparticles to photosensitizers has frequently been proposed as a key pathway, emerging evidence suggests that this may not be the predominant mechanism—particularly at the keV energy levels commonly employed in biomedical contexts. For example, Bulin et al. demonstrated through Monte Carlo simulations that a substantial portion of the incident photon energy is deposited locally within and around the nanoparticles.^40^ However, the subsequent energy relaxation cascade following ionizing radiation results in considerable losses in light emission efficiency, thereby reducing the effectiveness of the photodynamic response.^40^ These findings are consistent with the work of Secchi et al.,^39^ who reported that nonradiative energy transfer had a negligible impact on the X-ray activation of a photosensitizer bound to a chrysolite nanotube. Collectively, these insights support the hypothesis that singlet oxygen production and subsequent radiosensitization are driven by multiple, possibly synergistic, mechanisms. These include direct dose enhancement, water radiolysis, stochastic interactions between secondary radiation and the photosensitizer, and energy transfer. In this context, having demonstrated energy transfer from GdF_3_:Eu to the methylene blue (MB) shell (Figure 4), we proceeded to conduct cell-based experiments using both bare GdF_3_:Eu and the functionalized GdF@4B nanoparticles. The ensuing discussion focuses on disentangling the respective contributions of the GdF_3_:Eu core and the MB surface functionalization to the overall therapeutic efficacy.

### 3.3 Cell Viability

MTT assays without X-ray exposure were used to investigate the inherent toxicity of GdF_3_:Eu bare and GdF@4B in A549 (human) and LLC mice lung cancer cells (Figure 6a,b), determining the IC50 by nonlinear fitting. For the A549 cells, the IC50 was 1.6 mg/mL for bare GdF_3_:Eu and 1.5 mg/mL for GdF@4B. However, the viability profile for LLC cells presented a different behavior, with GdF@4B being much more toxic than the bare GdF_3_:Eu nanoparticle. The IC50 was 1.2 mg/mL for pure GdF_3_:Eu and 0.16 mg/mL for GdF@4B.

**Figure 6:**
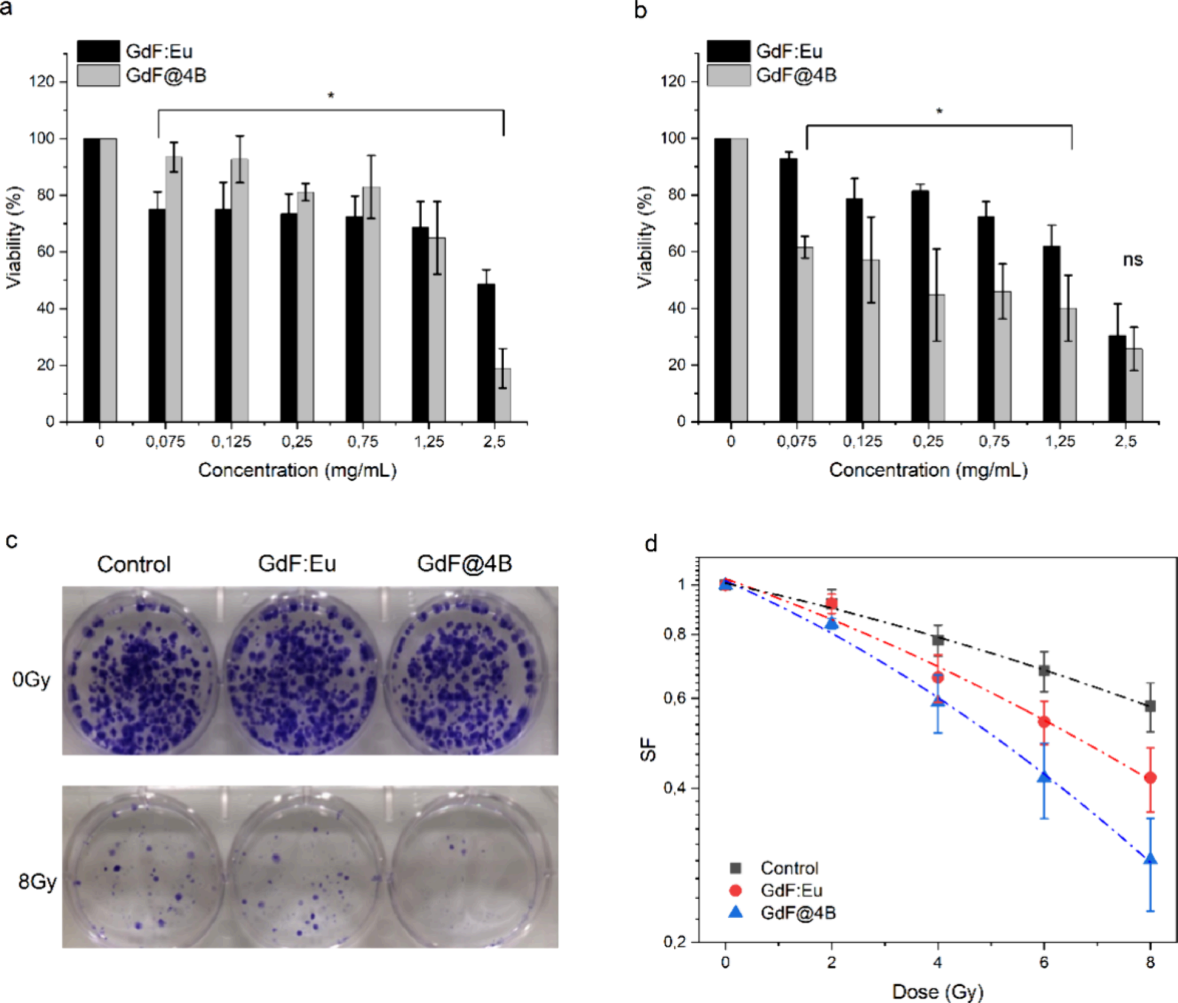
MTT assay results for bare GdF_3_:Eu and GdF@4B nanoparticles without X-ray irradiation in (a) A549 cells and (b) LLC cells. (c) Clonogenic assay plates for control (no nanoparticles), bare GdF_3_:Eu, and GdF@4B nanoparticles. (d) Survival fraction curves of X-ray-irradiated cells (220 kVp, 0–8 Gy) for control, bare GdF_3_:Eu, and GdF@4B nanoparticles.

The primary distinction between LLC and A549 cells lies in their species origin—LLC cells derive from a murine lung carcinoma model, whereas A549 cells originate from human lung tissue. A key difference between mouse and human cells is their oxidative stress response.^41^ Studies suggest that mouse embryonic fibroblasts (MEFs) exhibit higher sensitivity to oxygen levels, accumulating more DNA damage in higher oxygen conditions compared to human fibroblasts.^42^ Additionally, mitochondrial ROS generation, which influences oxidative stress responses,^43^ is lower in long-lived species like humans than in shorter-lived ones like mice.^44^ Moreover, compared to human cells, mouse cells excrete 18 times more DNA degradation products under the action of endogenous oxidants.^45^

LLC and A549 cells also differ in nanoparticle internalization. Research using camptothecin-loaded manganese dioxide-coated polydopamine nanoparticles revealed greater uptake in LLC cells compared to A549 cells (Figure 4),^46^ suggesting variations in endocytic pathways or lysosomal processing, which can affect nanoparticle-mediated ROS responses.^47^ Furthermore, TiO_2_ nanoparticles generate singlet oxygen (^1^O_2_) during photocatalytic reactions as well.^48,49^ WST-1 cell viability assay with different concentrations of TiO2 in the absence of irradiation showed that there was a concentration-dependent decrease in cell viability at 24 h via activating the Caspase 3-dependent apoptotic cell death.^50^ These factors may contribute to the differential toxicity observed following GdF@B treatment in A549 and LLC cells. While further mechanistic studies are a logical next step, they fall beyond the scope of this publication and are planned for future research.

Cells were treated with GdF_3_:Eu and MB-conjugated nanoparticles to evaluate their intrinsic cytotoxicity. Initially, the nanoparticles were dispersed in nonsupplemented RPMI 1640 medium at a concentration of 5 mg/mL. Subsequently, working concentrations were prepared by diluting this stock dispersion in RPMI 1640 medium supplemented with 10% fetal bovine serum (FBS) and antibiotics and used to treat the cells. Although the treatment was carried out in supplemented medium, the initial dispersion step in the absence of serum aimed to preserve the intrinsic characteristics of the nanomaterials before exposure. Serum proteins are known to adsorb onto nanoparticle surfaces, forming a protein corona that can alter their physicochemical properties and biological interactions. Therefore, understanding the colloidal stability of the nanoparticles in biological media is essential.

To evaluate nanoparticle behavior under physiological-like conditions, DLS measurements were performed for nanoparticles suspended in both ultrapure water and complete RPMI medium (Figure S6). Bare GdF_3_:Eu nanoparticles showed an approximate 50% increase in hydrodynamic diameter in the supplemented medium, likely due to aggregation resulting from electrostatic instability caused by the medium exchange. In contrast, GdF@4B nanoparticles exhibited no significant size change, indicating enhanced colloidal stability due to surface modification.

The preserved colloidal stability of GdF@4B in complete medium supports the reliability of cytotoxicity results obtained using serum-containing conditions. It also suggests that minimal aggregation occurred and that the observed cellular effects can be attributed primarily to the nanoparticles themselves rather than to secondary effects from aggregation. Thus, the experimental design allows for a meaningful evaluation of the intrinsic cytotoxic potential of the nanoparticles while also reflecting physiologically relevant conditions.

The clonogenic assay evaluated the reproductive capacity of cells exposed to X-rays (220 kVp). However, for LLC cells, even at a concentration of 0.075 mg/mL and without irradiation, there was no cell growth in the samples with GdF@4B, further indicating that mouse cells are more vulnerable, as already noted by the MTT assay. Thus, the clonogenic assay was performed with A549 cells at 0.075 mg/mL, since for GdF_3_:Eu bare nanoparticle, the viability indicated by the MTT assay was around 80%. After X-ray irradiation (8Gy), the number of colonies in whole samples was diminished (compared to nonirradiated samples) due to the natural effect of ionizing radiation. Still, the plates averaged fewer colonies for cells treated with GdF@4B and X-ray (Figure 6c). Another significant result was observed for the nonirradiated condition, in which there are no differences between the amount of colonies for the samples, indicating that, in the absence of X-rays, both nanoparticles tested (bare GdF_3_:Eu and GdF@4B) are safe for cell treatment.

To understand the effect of X-rays on the reproductive capacity of the cells, a graph of the survival fraction as a function of dose was plotted (Figure 6d). A statistical analysis (two-way ANOVA) to assess the significance of the results was performed using the p-value, calculated from the deviation between the observed value and the chosen reference value. A *p*-value ≤ 0.050 is generally considered statistically significant.^51^ Comparing the survival fraction curve for the control sample and the GdF_3_:Eu group, the value obtained was *p* = 0.0018; for bare GdF_3_:Eu and GdF@4B, the value was *p* = 0.0022, and for the control and GdF@4B was *p* = 0.0010. Thus, there is a significant difference between the survival fraction curves for all groups with *p* < 0.005.

The survival fraction was calculated using models already established in the literature.^52,53^ The X-ray interaction is enhanced with the high atomic number of gadolinium (*Z* = 64), resulting in the emission of a large amount of low-energy electrons (photoelectrons and Auger electrons) and, consequently, more significant cell damage. Consequently, the cell exposed to nanoparticles (with or without MB) has a lower survival fraction than the control group. However, the cells with GdF@4B had the lowest survival fraction curve because, in addition to the increased atomic number, previous results showed that the scintillation of GdF_3_:Eu excites MB, producing ^1^O_2_. Therefore, this result reveals that the ^1^O_2_ production, as demonstrated by the TPC-ESR experiments, is enough to cause a significantly smaller survival fraction of cancer cells.

The survival fraction curve was fitted using the linear-quadratic model:

$$\mathrm{SF}=e^{-\alpha D-\beta D^{2}}$$

where the α parameter reflects the component of cell reproductive death that is linearly proportional to the dose, whereas β represents the component that is proportional to the square of the dose. The linear-quadratic model is initially dominated by the linear term α, which is sensitive to low doses, followed by an increased curvature as the quadratic term β becomes more significant.

The reproductive integrity of the cell is correlated to the frequency of chromosomal aberrations, especially exchange-type aberrations, which require double-strand breaks (DSB). At low doses, DSB is more likely to be caused by the same electron. Then, the probability of aberration is proportional to the dose (D). Consequently, the survival curve is linear at low doses, represented by the linear term α. At higher doses, DSBs become more likely due to the striking of different electrons and, in this case, the probability of interaction between the two breaks is proportional to the square of the dose, represented by the term β.^54^ Thus, cells with a high α/β ratio exhibit a relatively constant rate of cell reproductive death with increasing dose, while curves with a low α/β ratio show a pronounced curvature.^52^ Table 1 summarizes the results of α and β for the tested NPs (Control, bare GdF_3_:Eu, and GdF@4B).

**Table 1:** Values of α, β, SER, and DEF for Nontreated Cells (Control) and for Cells Treated with Bare GdF_3_:Eu NPs, and GdF@4B^a^

	control	GdF_3_:Eu	GdF@4B	GdF@4B/GdF_3_:Eu
α	0.061	0.08	0.086	1.1
β	0.0013	0.0041	0.0097	2.4
α/β	46.2	19.6	8.9	0.5
SER	1	2.32	3.84	1.7
DEF (2Gy)	1	1.36	1.53	1.1
DEF (4Gy)	1	1.39	1.65	1.2
DEF (6Gy)	1	1.42	1.74	1.2
DEF (8Gy)	1	1.44	1.81	1.3

a The GdF@4B/GdF_3_:Eu column represent the ratio of the GdF_3_@4B parameters by the GdF_3_:Eu parameters.

The α terms for GdF_3_ and GdF@4B are around 30 and 40% higher compared to the control. This increase in the α term is, however, small compared to the increase observed for the β term. In this case, the increase in the β term for GdF_3_ relative to the control was 3.2 times, which may be associated with the high atomic number of the NP, increasing X-ray interaction with ionizing radiation, and producing more low-energy electrons in the medium. For the cells treated with GdF@4B, the β term enhancement was 7.5 times, likely explained by the increased ^1^O_2_ production, which also causes DNA and cell damage.^7^ Here, it is worth noting the synergistic effect of the high atomic number of the GdF_3_ cores associated with ^1^O_2_ production allowed by the ScNP@photosensitizer conjugates.

Considering the radiosensitization response of the GdF@4B group is influenced by both the atomic number and ^1^O_2_ production, we also calculated the GdF@4B/GdF_3_:Eu parameters ratio, aiming to elucidate the real influence of the ^1^O_2_ production (Table 1). The fifth column in Table 1 clearly depicts that the α term increases by only 10% while the β term increases by 240%. As a consequence, the α/β falls to half. Once the ^1^O_2_ production increases upon augmenting the dose, as revealed by Figure 5, the SF fraction will decrease faster at higher doses due to the increased ^1^O_2_ concentration. As a consequence, the SF curve becomes steeper, resulting in increased values of the β term. The damage caused by ^1^O_2_ is generally related to secondary factors such as the induction of oxidative stress, which, in turn, triggers the apoptosis process.^7^ Additionally, ^1^O_2_ contributes to the inhibition of the Bcl-2 protein and increased expression of the BAX protein, increasing the permeability of the outer mitochondrial membrane and triggering processes that lead to apoptosis via DNA fragmentation and cellular protein degradation.^7^ Other mechanisms of interaction between ^1^O_2_ and cells may also lead to cell death.

In addition to the analysis of the α and β parameters, other factors can be calculated for a more comprehensive evaluation, as summarized in Table 1, for the NPs tested in this study. One such factor is the **Sensitization Enhancement Ratio (SER)**, which indicates the radiobiological impact of the NP compared to the nonexposed group. The SER is calculated by the ratio of the total area under the SF curve of the control group to the total area under the SF curve of the NP group. Cells treated with GdF@4B presented a much higher SER value compared to those of the cells treated with bare GdF_3_:Eu and the control. In other words, the GdF@4B NPs are able to increase radiosensitization by 3.8-fold. Considering the GdF@4B/GdF_3_:Eu SER ratio of 1.7 (5th column in Table 1), we infer that 70% of the SER = 3.8 obtained for the GdF_3_@4B group could be attributed to the ^1^O_2_ sensitization. Therefore, according to the SER parameter, although ^1^O_2_ production dominates cell radiosensitization over high Z, the latter still has an important contribution to the SF, emphasizing the importance of the synergic effect of high Z and ^1^O_2_ production. Here, it should be emphasized that the individual contribution of the atomic number and ^1^O_2_ generation may drastically change with the ionizing radiation type and energy. Moreover, once the ^1^O_2_ generation is usually proportional to the scintillation intensity, it will depend on the atomic number of the core as well.

Another analyzed parameter was the **Dose Enhancement Factor (DEFxGy)**, defined for a specific dose (xGy) as the ratio of the dose in the sample with NPs (D_NP_) that produces the same survival fraction as the dose xGy in the control sample (DEF_xGy_ = $\frac{\mathrm{xGy}}{D_{\mathrm{NP}}}$ ). In other words, the higher the DEF value, the lower the dose required in the NP sample (D_NP_) to produce the same SF as the control group. For the GdF@4B NPs, the DEF value increases from 1.53 up to 1.81 (18% increase) in the 2–8 Gy dose range, indicating that cells treated with GdF@4B require a lower dose to achieve the same SF. Another interesting result is that the DEF increase as a function of dose is much more consistent for the GdF@4B group (18% increase) compared to that of the GdF_3_:Eu group (7% increase). Looking to the GdF@4B/GdF_3_:Eu _3_ DEF ratio (5th column, Table 1), we once again infer that the ^1^O_2_ contribution to the SF becomes more relevant at higher doses.

Although there is no consensus, some researchers suggest that DEF at a dose of 2 Gy (DEF_2Gy_) is the most relevant parameter since 2 Gy is typically used in conventional fractionated radiotherapy. However, it is important to point out that, despite the observed trend of higher DEF upon increasing the dose, the DEF_2Gy_ for the GdF_3_@4B is already significantly high (1.53), revealing that, in a fractionated RT protocol, cells treated with GdF_3_@4B would require a fraction dose around 1.3 Gy to achieve the same SF of nontreated cells receiving a 2 Gy fraction dose, decreasing the dose necessary to treat the tumor and, consequently, the immediate and late effects of radiation. Moreover, the increased radiosensitization at higher doses suggest that the GdF_3_@4B NPs may be a promising treatment option combined to radiosurgery, a more advanced RT modality, which employs a single high dose to treat cancer (instead of several small doses employed in the conventional fractionated RT) and, therefore, could be further benefited by the increased ^1^O_2_ concentration obtained at the higher doses.

In vivo studies evaluating the biodistribution, safety, and therapeutic efficacy of nanoplatforms are essential to support their clinical translation. The work by Sun et al. demonstrated that NaGdF_4_:Eu^3^^+^ nanoparticles exhibit selective tumor accumulation, low systemic toxicity, and significant tumor growth inhibition upon radiation activation.^55^ These findings highlight the importance of experimentally validating therapeutic nanostructures and can be directly translated to analogous platforms, such as GdF:Eu nanoparticles functionalized with MB, particularly in the context of combined therapies such as X-PDT.

## 4 Conclusions

In this work, we successfully synthesized and characterized GdF_3_:Eu nanoparticles using a microfluidic reactor, optimizing their conjugation with MB for X-PDT. The conjugation process with MB altered the size, polydispersity, and surface charge of the GdF_3_:Eu nanoparticles, indicating a decreased stability at higher MB concentrations. The combination of GdF_3_:Eu scintillation properties and efficient energy transfer to the photosensitizer MB resulted in an effective ^1^O_2_ production under X-ray irradiation. Our findings emphasize the critical role of surface control and precise conjugation in maximizing ROS generation and therapeutic efficacy. ^1^O_2_ production was investigated for varying energy beams and radiation doses, revealing a higher ^1^O_2_ generation for lower energies and higher doses. The dose-dependent ^1^O_2_ generation translated into increased radiosensitization at higher doses, as suggested by all radiosensitization parameters obtained from the clonogenic assay survival curves. Despite the observed trend of higher DEF upon increasing the dose, the DEF_2Gy_ for the GdF_3_@4B is high enough (1.53) to significantly decrease the dose necessary to treat the tumor and, consequently, the immediate and late effects of radiation. Therefore, GdF_3_@4B NPs demonstrated promising application for targeted cancer therapy, utilizing deep-tissue X-ray penetration and localized ROS production to enhance treatment precision and effectiveness in either conventional fractionated radiotherapy or modern radiosurgery treatments. Future studies should explore in vivo applications to further assess the system’s clinical potential and safety profile.

## Supplementary Material



## Notes

Radioluminescence (Figure S1) and photoluminescence (Figure S2) spectra obtained immediately after the synthesis and after 1 week; optical characterization of the nanoparticles and of the methylene blue to depict the good spectral overlap between nanoparticle emission and MB absorption/excitation (Figure S3); estimation of MB concentration adsorbed onto the nanoparticle’s surface (Figure S4); and thermogravimetric analysis (Figure S5) (PDF)
