# Supplementary material for: Enhanced Cancer Radiosensitization via Energy Transfer from Eu-Doped GdF3 Nanoparticles to Methylene Blue in X‑ray Photodynamic Therapy
Source: ACS Appl Mater Interfaces. 2025 Sep 4;17(37):51620–33. doi: 10.1021/acsami.5c10506 (PMC12447382; doi:10.1021/acsami.5c10506)
Supplement: Supplementary file 1 [file am5c10506_si_001.pdf]

Supporting Information

Enhanced Cancer Radiosensitization via Energy Transfer from Eu-Doped GdF_3 Nanoparticles to Methylene Blue in X-Ray Photodynamic Therapy

Mileni M. Isikawa[a], Zeinaf Muradova[b], Toby Morris [b,c], João V.V. Lessa[a], Fernanda H. Borges[d], Rógeria R. Gonçalves[d], Ross I. Berbeco[b], Eder J. Guidelli*[a]

[a] Departamento de Física - FFCLRP- Universidade de São Paulo, Ribeirão Preto, SP, Brasil

[b] Department of Radiation Oncology, Brigham and Women's Hospital, Dana-Farber Cancer Institute, Harvard Medical School, Boston, MA USA

[c] Department of Physics and Applied Physics, University of Massachusetts Lowell, Lowell, MA, USA

[d]Departamento de Química – FFCLRP, Universidade de São Paulo, Ribeirão Preto, SP 14040-901, Brazil

Corresponding author: Tel: +55 (16) 3315-0080 E-mail address: guidelli@usp.br **(E. J. Guidelli)**

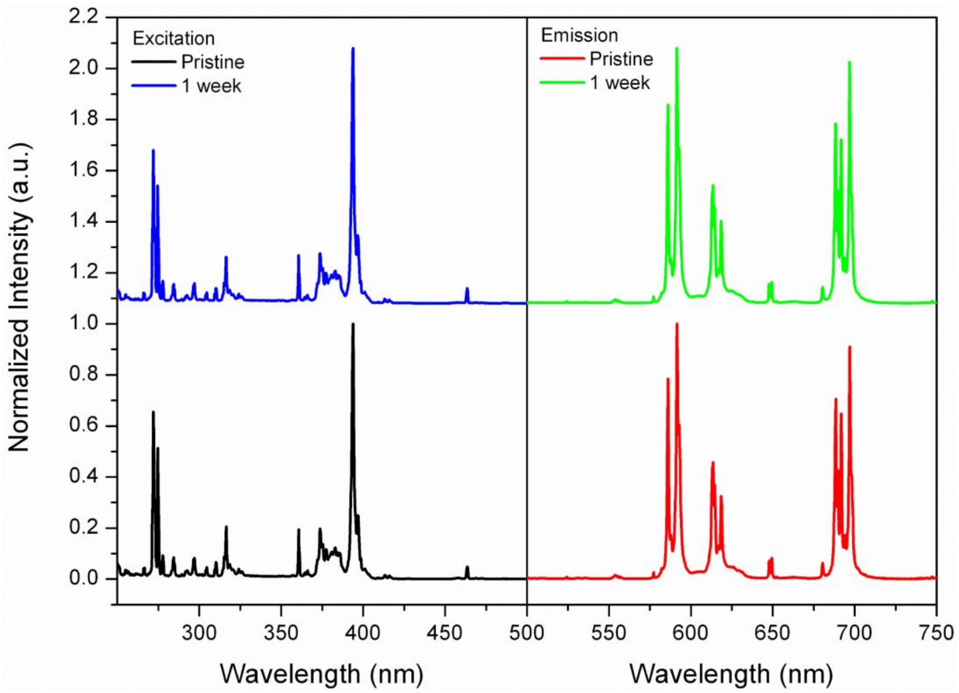

Figure S1: Photoluminescence excitation (emission fixed at 592 nm) and emission (excitation fixed at 394 nm) spectra collected from GdF_3:Eu powder samples immediately after the synthesis (as prepared samples - pristine) and after one week. The GdF_3:Eu was produced with 1 min reaction time and 10%mol Eu^{3+}.

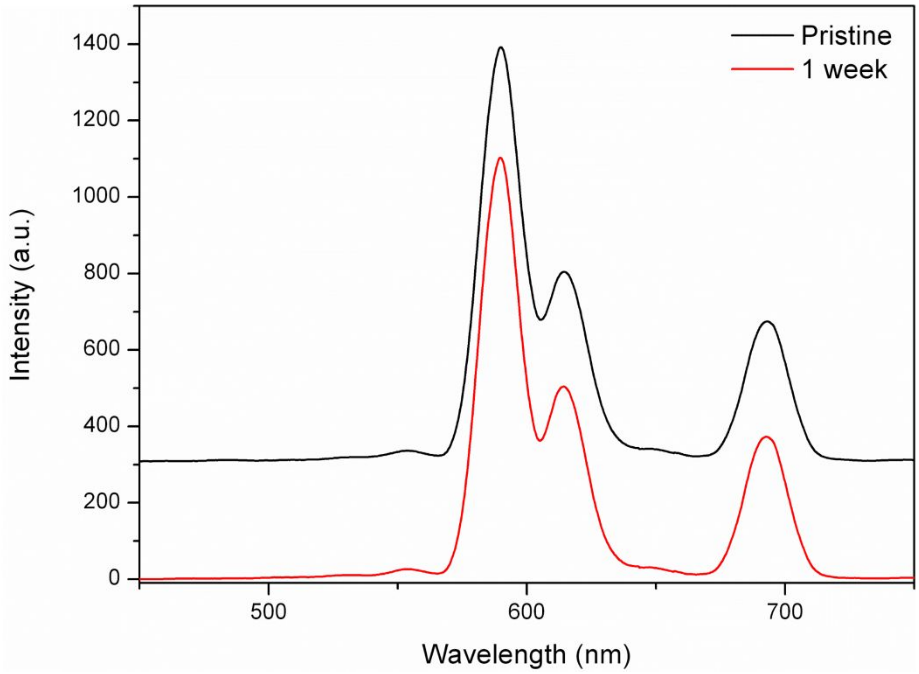

Figure S2: Radioluminescence spectra (scintillation) from GdF_3:Eu powder samples collected immediately after the synthesis (as prepared samples - pristine) and after one week. The GdF_3:Eu was produced with 1 min reaction time and 10%mol Eu^{3+}.

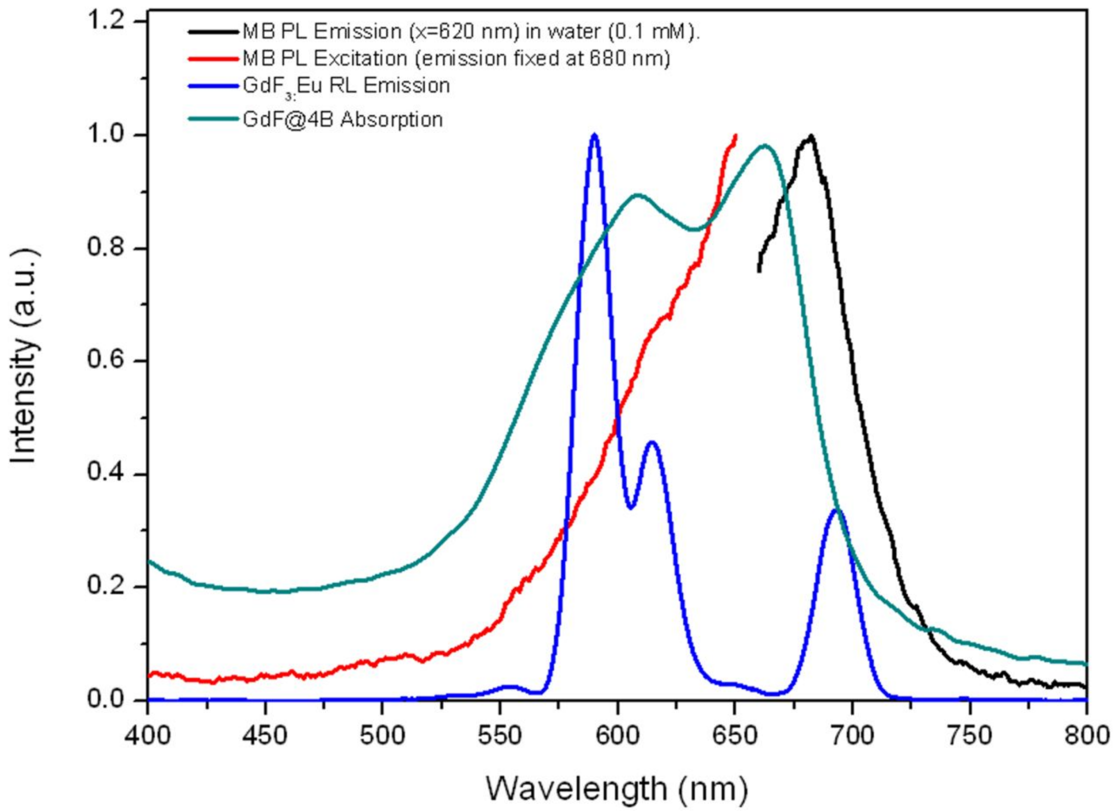

Figure S3: Methylene blue excitation (red) and emission (black). For the emission spectrum, excitation was fixed at 620 nm. For the excitation spectrum, emission was fixed at 680 nm. Both PL excitation and emission were collected for MB water solutions at 0.1 mM. The PL excitation spectrum reveal the presence of MB monomers when in water solution due to the absence of a significative shoulder at ~600 nm. The scintillation emission spectra of the GdF_3:Eu is plotted together to demonstrate the overlap between the nanoparticle emission and MB excitation. The absorbance of the GdF_3:Eu covered with 4 depositions cycles of MB is also shown to further demonstrate the good spectral overlap. It is worth noting that the MB PL excitation spectrum in solution does not exactly match the GdF@4B absorption spectrum due to dimer formation during the layer-by-layer shell deposition process, as evidenced by the significative shoulder ~ 600 nm.

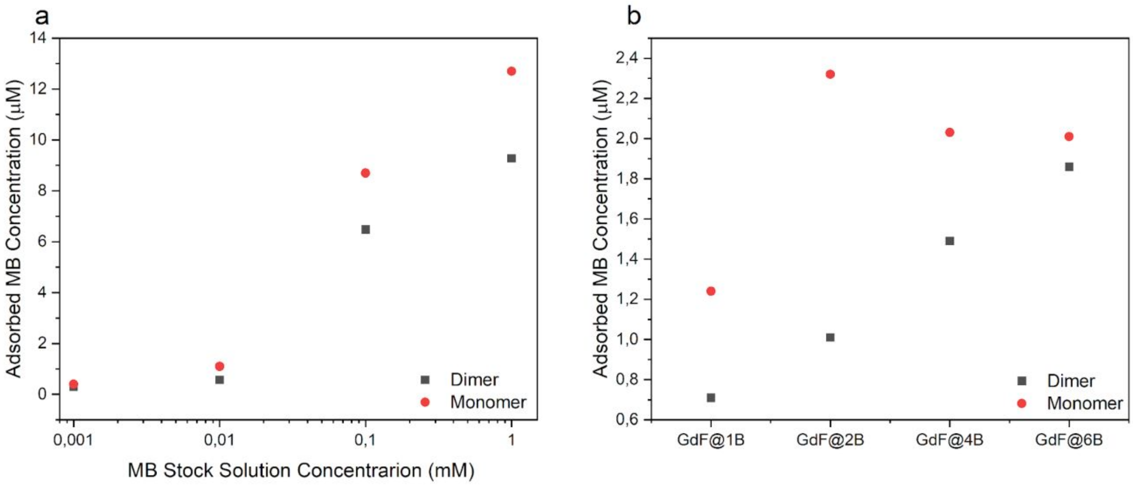

Figure S4: Estimation of MB concentration adsorbed on the (GdF_3:Eu) nanoparticle, (a) as a function of the MB concentration used during the deposition process and (b) the number of deposition cycles performed.

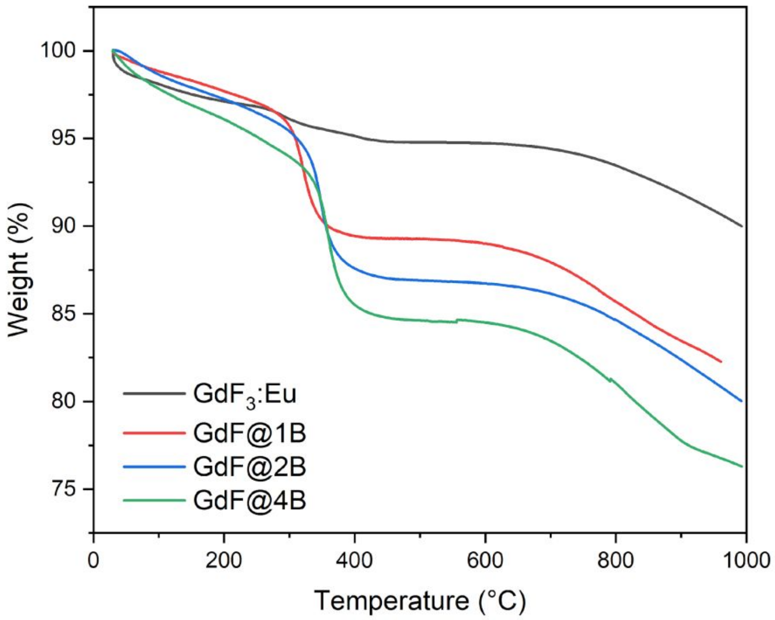

Figure S5: Thermogravimetric analysis (TGA) curves of bare GdF_3:Eu nanoparticles and samples with increasing number of MB deposition cycles: GdF@1B, GdF@2B, and GdF@4B. The progressive increase in mass loss corresponds to higher organic content adsorbed onto the nanoparticle's surface.

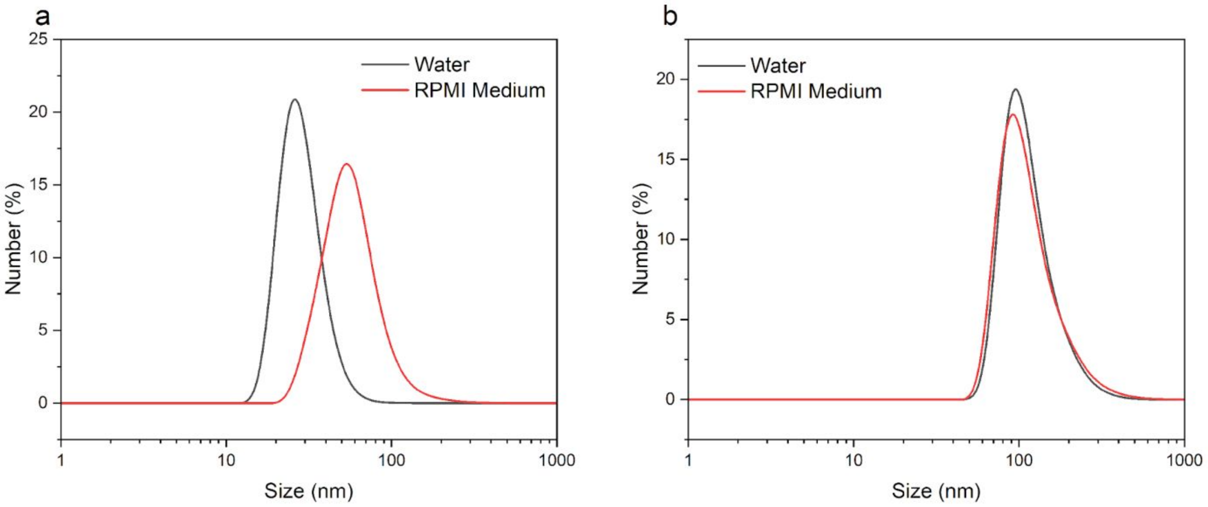

Figure S6: DLS analysis of the nanoparticles in water and RPMI culture medium for (a) pure GdF_3:Eu and (b) GdF@4B.
